# Supplementary material for: SYNERGISTIC ON AUXIN AND CYTOKININ 1 positively regulates growth and attenuates soil pathogen resistance
Source: Nat Commun. 2020 May 1;11:2170. doi: 10.1038/s41467-020-15895-5 (PMC7195429; doi:10.1038/s41467-020-15895-5)
Supplement: Supplementary file 1 — Supplementary Information [file 41467_2020_15895_MOESM1_ESM.pdf]

Supplementary information for manuscript

***Synergistic on auxin and cytokinin 1* positively regulates growth and attenuates soil pathogen resistance**

Andrej Hurný[1], Candela Cuesta[1,2$], Nicola Cavallari[1$], Krisztina Ötvös[1,3], Jerome Duclercq[4], Ladislav Dokladal[5,6], Juan Carlos Montesinos[1], Marçal Gallemí[1], Hana Semerádová[1], Thomas Rauter[1,7], Irene Stenzel[8], Geert Persiau[9,10], Freia Benade[11], Rishikesh Bhalearo[12], Eva Sykorova[5], András Gorzsás[13], Julien Sechet[14], Gregory Mouille[14], Ingo Heilmann[8], Geert De Jaeger[9,10], Jutta Ludwig-Müller[11], and Eva Benková[1*]

[1]*Institute of Science and Technology, Austria, Klosterneuburg.*
[2]*Instituto Universitario de Biotecnología de Asturias (IUBA). Departamento de Biología de Organismos y Sistemas, Universidad de Oviedo, Spain.*
[3]*AIT Austrian Institute of Technology, Center for Health & Bioresources. Bioresources Unit, Center for Health & Bioresources, AIT Austrian Institute of Technology GmbH, Tulln, Austria*
[4]*Unité 'Ecologie et Dynamique des Systèmes Anthropisés' (EDYSAN UMR CNRS 7058 CNRS), Université du Picardie Jules Verne, UFR des Sciences, Amiens, France.*
[5]*Institute of Biophysics, The Czech Academy of Sciences, Královopolská 135, 61265, Brno, Czech Republic.*
[6]*Mendel Centre for Plant Genomics and Proteomics, CEITEC, Masaryk University, Brno, Czech Republic*
[7]*Molecular Biology and Biochemistry, Gottfried Schatz Research Center, Medical University of Graz, Neue Stiftingtalstraße 6/6, 8010 Graz, Austria*
[8]*Department of Cellular Biochemistry, Institute for Biochemistry and Biotechnology, Martin-Luther-University Halle-Wittenberg, Halle, Germany.*
[9]*Department of Plant Biotechnology and Bioinformatics, Ghent University, Ghent, Belgium.*
[10]*VIB Center for Plant Systems Biology, Ghent, Belgium.*
[11]*Institut für Botanik, Technische Universität Dresden, Dresden, Germany.*
[12]*Umeå Plant Science Centre, Department of Forest Genetics and Plant Physiology, Swedish University of Agricultural Sciences, S-901 83 Umeå, Sweden.*
[13]*Department of Chemistry, Umeå University, Linnaeus väg 6, SE-901 87 Umeå, Sweden.*
[14]*Institut Jean-Pierre Bourgin, INRAE, AgroParisTech, Université Paris-Saclay, 78000, Versailles, France*

[$] These authors contributed equally

*Correspondence: eva.benkova@ist.ac.at

List of supplementary material:

Supplementary figures 1-7

Supplementary table 1

Supplementary dataset 1

Supplementary dataset 1

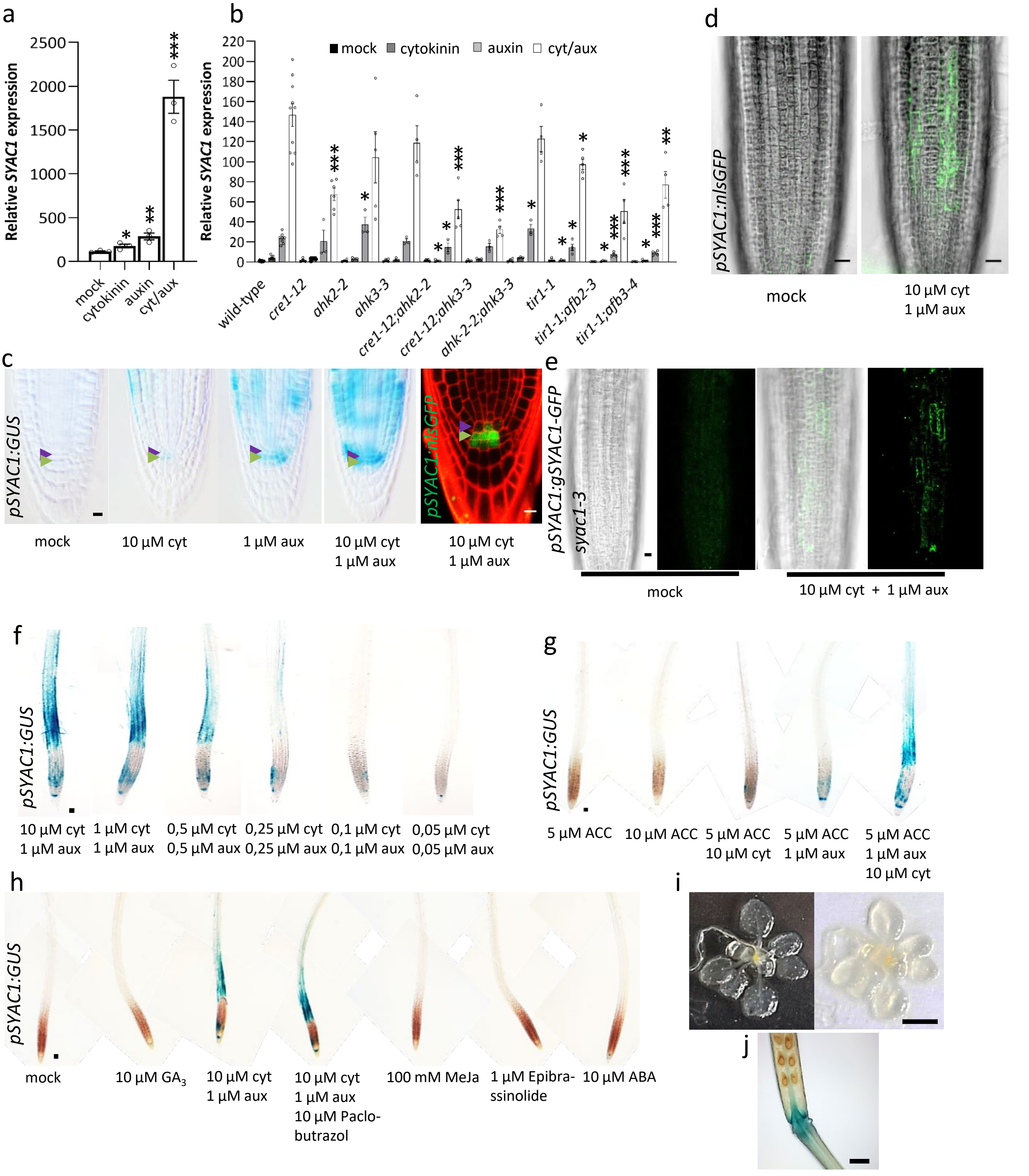

Supplementary Figure 1. Expression pattern of *SYAC1* in response to hormonal treatments. (a, b) Realtive *SYAC1* expression in roots of 5-day-old wild-type (a); wild-type, cytokinin (*ahk2-2*, *ahk3-3*, and *cre1-12/ahk4)* and auxin (*tir1-1*; *tir1-1,afb2-3* and *tir1-1,afb3-4)* single and multiple receptor mutants (b) treated with 10 µM cytokinin, 1 µM auxin or both hormones together for 3 hours detected by transcriptome profiling (a) or by RT-qPCR (b). Significant differences when compared to mock treated roots (a) and respective treatment of wild-type (b) are indicated as *P < 0.05, **P < 0.01 and ***P < 0.001 (*t* test, n=5 biological replicates with 3 technical replicates each, average ± SE). (c-e) Expression of *pSYAC1:GUS* (c) and *pSYAC1:nlsGFP* (c, d) and *pSYAC1:gSYAC1-GFP syac1-3* (e) in 5-day-old root tips treated with mock, auxin and cytokinin for 6 hours. Purple and green arrowheads indicate the quiescent center (QI) and columella initials (CI), respectively. (f-h) *pSYAC1:GUS* expression in roots treated with different concentrations of auxin and cytokinin (f); ethylene precursor 1-aminocyclopropane-1-carboxylic acid (ACC) in combination with auxin and cytokinin (g) and gibberellins (GA_3), inhibitor of gibberellin biosynthesis (paclobutrazol), methyl jasmonate (MeJa), brassinosteroids (Epibrassinolide) and abscisic acid (ABA) (h) for 6 hours. (i, j) *pSYAC1:GUS* expression not detected in rosette leaves of 3-week-old plants (i); strong GUS staining in abscission zone of silique from 8 week-old plant (j). Scale bar 10µm (c, e), 20 µm (d), 50µm (f-h), 500µm (i), 100µm (j). 1-Naphthaleneacetic acid and N6-benzyladenine used as auxin and cytokinin, respectively.

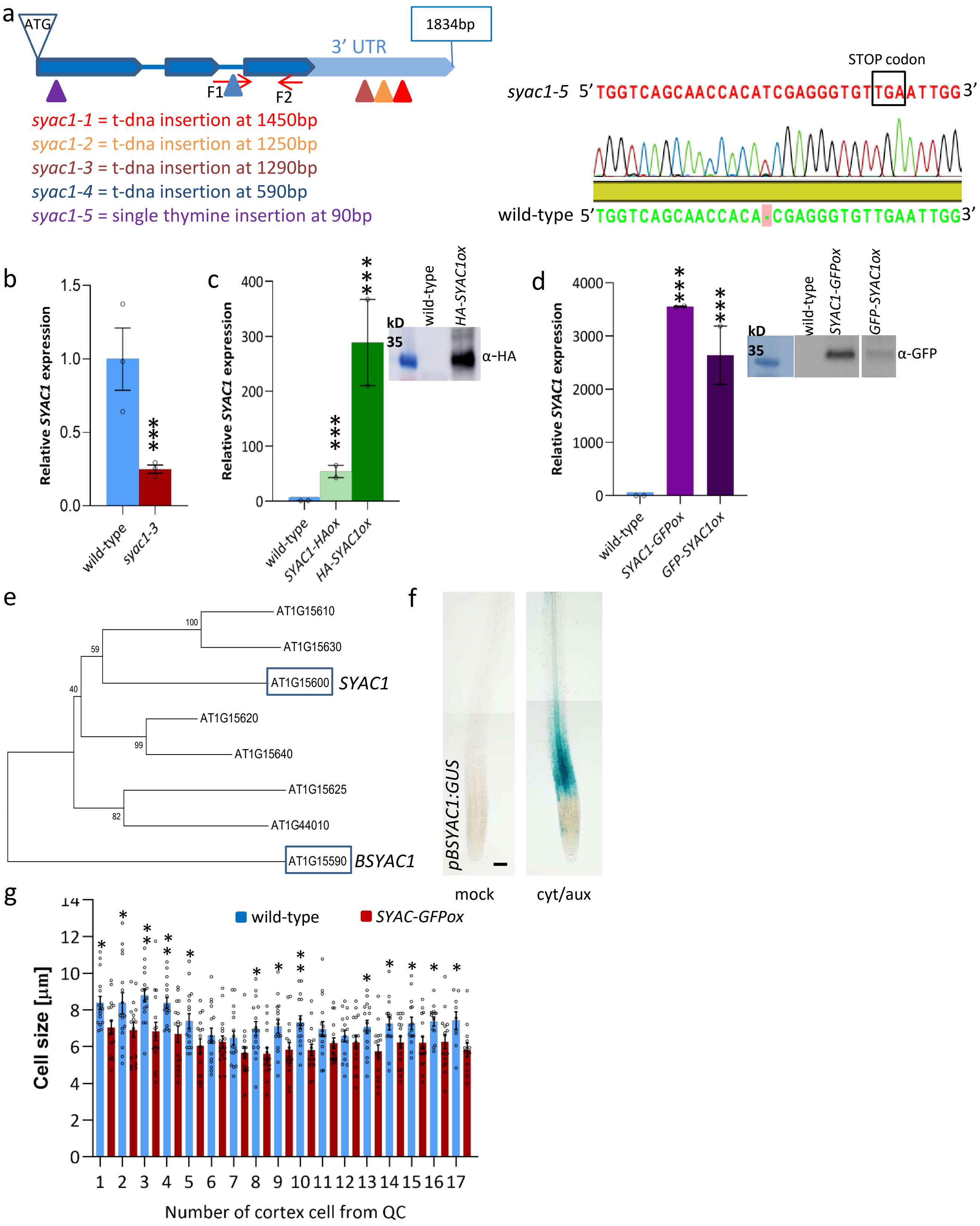

Supplementary figure 2. Isolation of *SYAC1* mutant alleles and overexpressor lines. (a) Exon/intron map of the *SYAC1* gene (from https://www.arabidopsis.org/, left panel), positions of T-DNA insertions and position of single nucleotide insertion in *syac1-5* CRISPR allele (right panel), which results in a premature stop codon. F1 and F2 arrows indicate the position of primers used for RT-qPCR expression analyses. (b-d) Relative expression of *SYAC1* in mutant and overexpressor (*SYAC1ox*) lines monitored by RT-qPCR (***P< 0.001 *t* test; n = 3 biological replicates with 3 technical replicates each, average ± SE). Expression of SYAC1 fusion constructs detected by Western blot analysis (insets) using α-HA and anti-GFP specific antibodies. (e) Phylogenetic tree of *SYAC1* related genes. (f) Expression of *pBSYAC1:GUS (AT1G15590)* in roots of 5-day-old seedlings treated with auxin (1 µM) and cytokinin (10 µM) for 6 hours. Scale bar 50 µm. (g) Size of cortex cells in roots of 5 day-old wild-type and *SYAC1-GFPox* seedlings. Significant differences are indicated as *P < 0.05 and **P < 0.01 (*t* test; n = 15-20 roots, average ± SE).

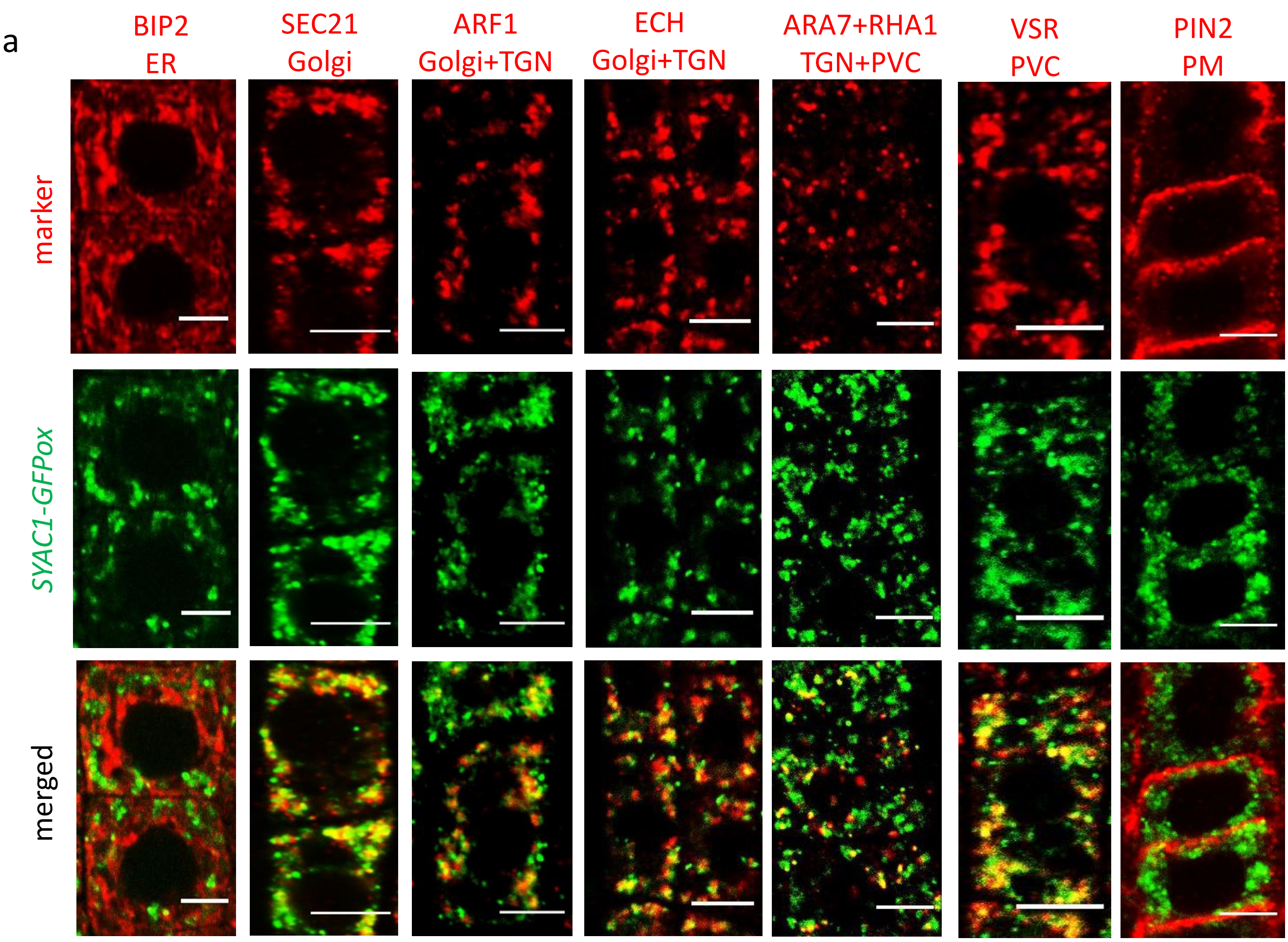

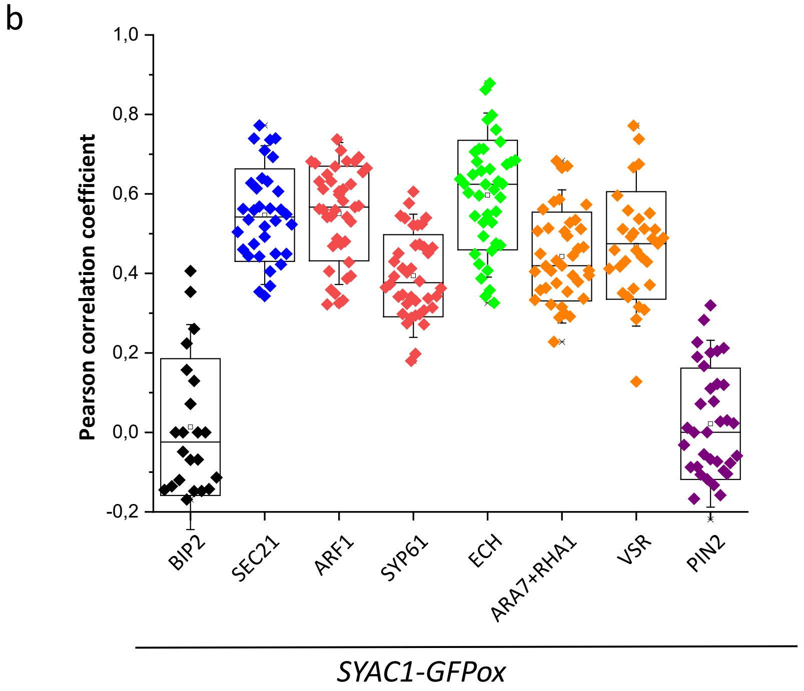

Protein	Localization
BIP2	ER
SEC21	Golgi
ARF1	Golgi+TGN
SYP61	Golgi+TGN
ECH	TGN
ARA7+RHA1	TGN+PVC
VSR1	PVC
PIN2	PM

c

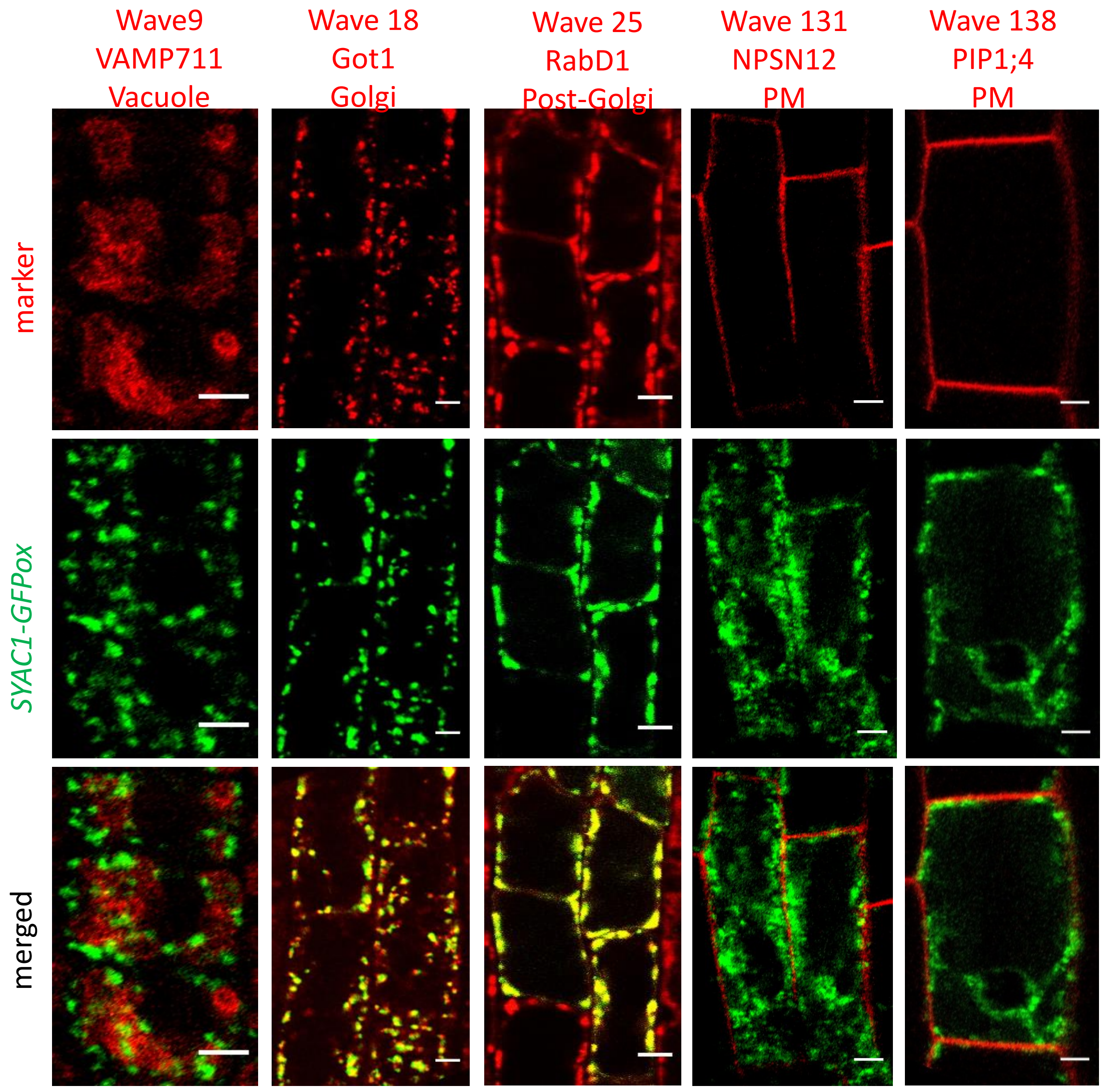

d

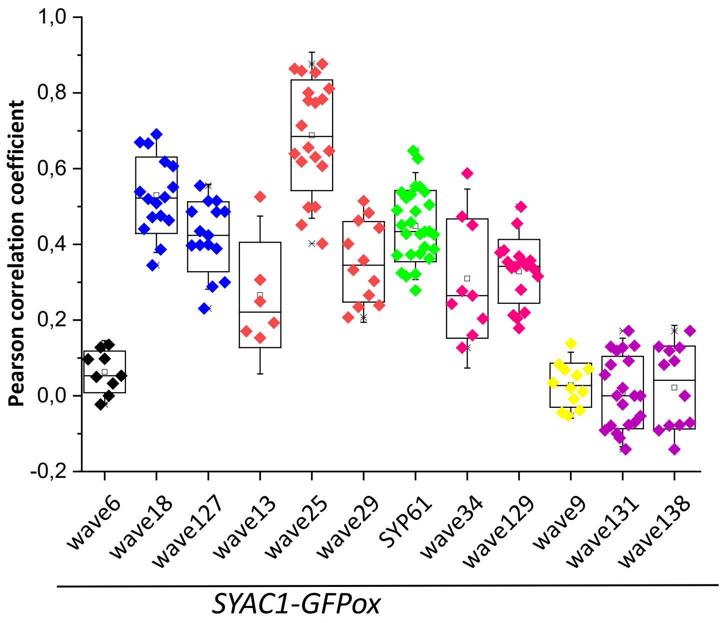

	Protein	Localization
wave6	NIP1;1	ER+PM
wave18	Got1	Golgi
wave127	MEMB12	Golgi
wave13	VTI12	TGN+EE
wave25	Rab D1	Post -golgi +endosomal
wave29	Rab D2a	Golgi+endosomal
SYP61	SYP61	TGN
wave34	Rab A1e	Endosomal+RE
wave129	RabA1g	Endosomal+RE
wave9	VAMP711	Vacuole
wave131	NPSN12	PM
wave138	PIP1;4	PM

Supplementary figure 3. SYAC1 co-localizes with Golgi/TGN/Endosomal/PVC markers. (a,c) Co-localization of SYAC1-GFP with markers for different subcellular compartments using antibodies against GFP and subcellular markers (a) and by co-expression with selected markers from collection of wave reporters [33]. (c). (b, d) Quantification of the SYAC1 co-localization with subcellular markers using Pearson correlation coefficient. 5 day-old seedlings of *SYAC1-GFPox* grown on mock medium used in co-localization experiments (n = 10 roots with 1-5 epidermal cells each). In the boxplots, center lines show the medians; box limits indicate the 25th and 75th percentiles as determined by Origin software; whiskers extend 1.5 times the interquartile range from the 25th and 75th percentiles, individual datapoints are represented by dots. ER-Endoplasmic reticulum, TGN-trans-Golgi network, PVC-prevacuolar compartment, PM-plasma membrane, EE-early endosomes, RE-recycling endosomes. Scale bar 5 µm.

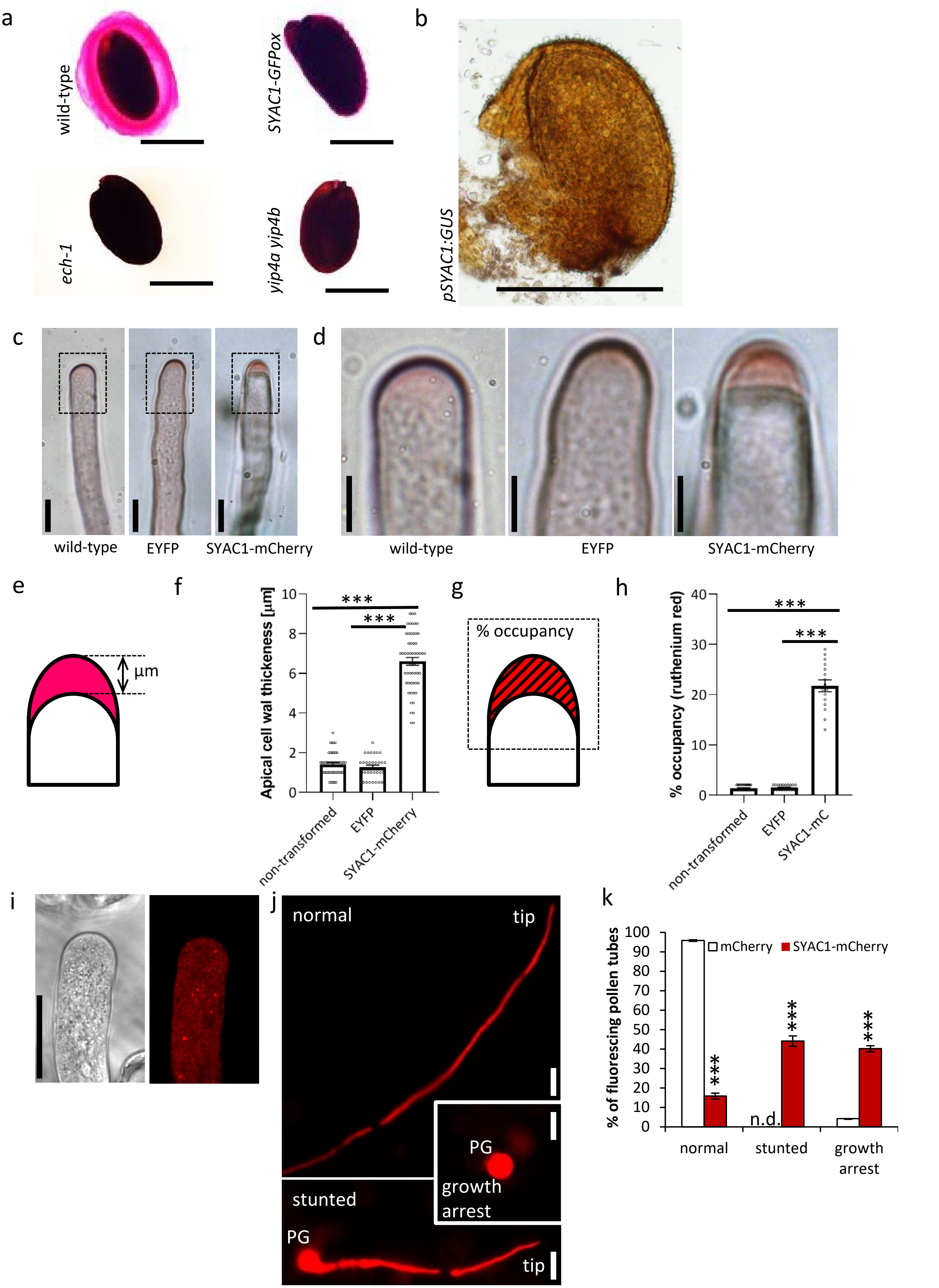

Supplementary figure 4. SYAC1 alters pectin secretion in *Arabidopsis* seeds and in tobacco pollen tubes (a) Ruthenium red–stained seed coat mucilage after imbibition of *wild-type*, *SYAC1-GFPox, ech-1* and *yip4a yip4b* seeds. Representative images shown (~100 seeds stained per line). Scale bar 200 µm. (b) Analysis of *pSYAC1-GUS* expression in seed coat. Scale bar 200 µm. (c-k) The effects of increased expression of SYAC1-mCherry on apical pectin deposition and pollen tube growth were tested upon transient expression in tobacco (*Nicotiana tabacum*) pollen tubes. (c, d) Pectin deposition in pollen tubes expressing SYAC1-mCherry, non-transformed controls (wild-type) or pollen tubes expressing an EYFP control was assessed by staining with ruthenium red. (c) Overview; scale bars 10 µm. Boxes, area magnified in (d). (d) Detail; Scale bars 5 µm.

(e, f) The thickness of the apical cell walls was determined in microscopic images as indicated in (e). (f) Apical cell wall thickness in non-transformed controls, in pollen tubes expressing an EYFP control or in cells expressing SYAC1-mCherry. Data are means ± SE from three independent experiments (non-transformed, 47 cells; EYFP, 31 cells; SYAC1-mCherry, 55 cells). (g, h) The extent of ruthenium red-staining was further assessed by determining the dye occupancy (hatched area) as a percentage of a square area around the tip, as indicated in (g). (h) Occupancy of ruthenium red in non-transformed controls, in pollen tubes expressing an EYFP control or in cells expressing SYAC1-mCherry. Data are means ± SE from two independent experiments (non-transformed, 35 cells; EYFP, 21 cells; SYAC1-mCherry, 16 cells). SYAC1-mCherry fusion protein was transiently expressed in tobacco (*Nicotiana tabacum*) pollen tubes by particle bombardment. (i) At low expression levels, the SYAC1-mCherry protein localized in dynamic punctate structures, consistent with an association with a transitory membrane compartment of the secretory pathway. Left, bright field image; right, SYAC1-mCherry fluorescence (false color). Scale bar 10 µm. (j) At intermediate and high levels, the expression of SYAC1-mCherry resulted in morphological changes in the pollen tube cells. These were characterized by a large proportion of stunted pollen tubes or the failure of pollen grains to produce a tube ("growth arrest"), as indicated. PG, pollen grain; tip, pollen tube tip. Scale bars, 40 µm. (k) The incidence of stunted and arrested pollen tubes was scored first in control pollen tubes expressing mCherry (white bars)and in tubes expressing SYAC1-mCherry (red bars). Scoring data are given for each morphological category (normal, stunted, growth arrest) as the mean percentages ± SE of the total number of recorded pollen tubes and were obtained in seven independent experiments with > 150 cells for each construct. n.d., not detected. Significant differences are indicated as ***P < 0.001 (*t* test).

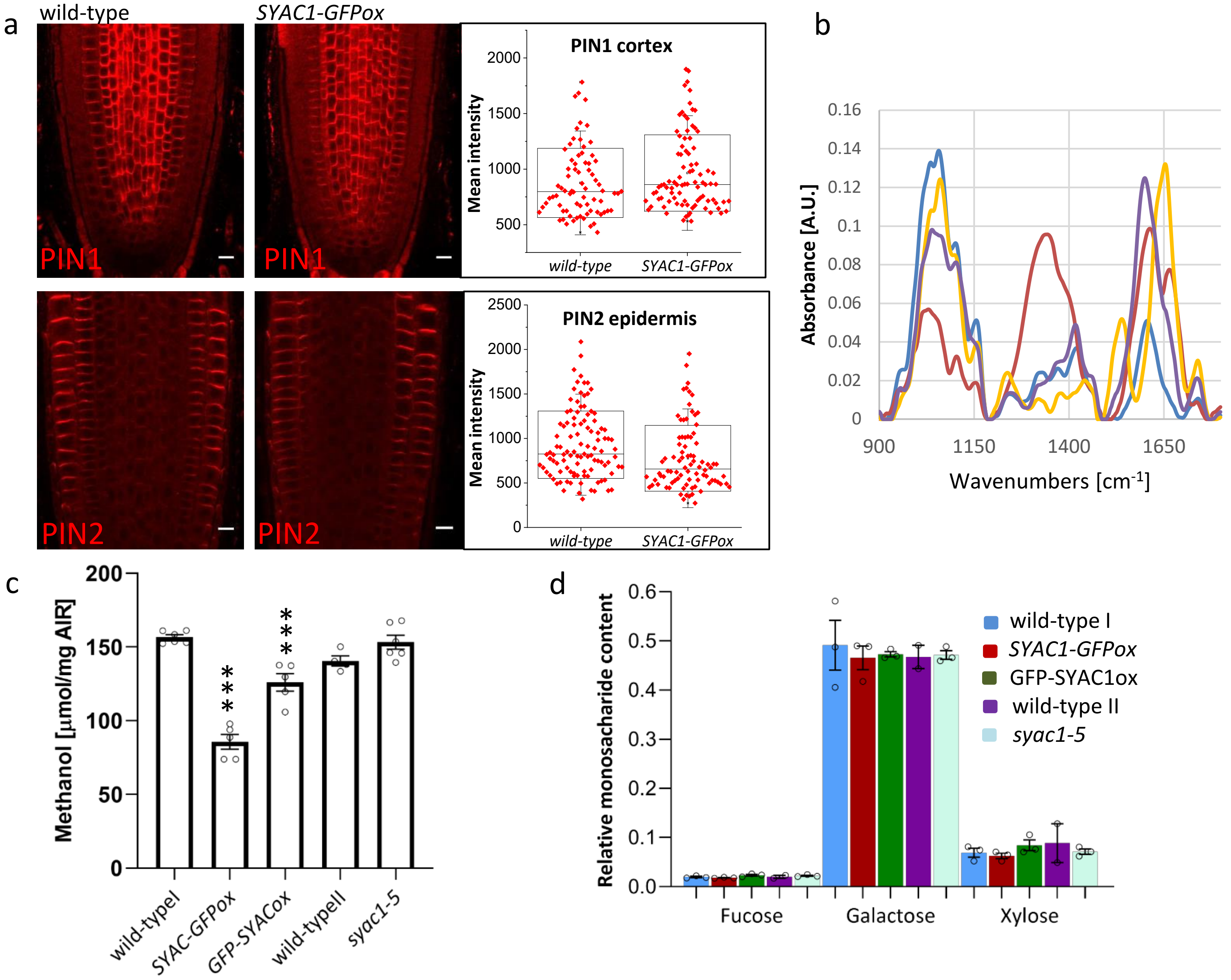

Supplementary figure 5. SYAC1 alters cell wall composition. (a) Immunolocalisation of PIN1 and PIN2 in roots of wild-type and *SYAC1-GFPox* using PIN1 and PIN2 specific antibodies. Scale bar 10 µm. Average of intensity at the plasma membrane measured in 5 -10 roots (10 cells for each). In the boxplots, center lines show the medians; box limits indicate the 25th and 75th percentiles as determined by Origin software; whiskers extend 1.5 times the interquartile range from the 25th and 75th percentiles, individual data points are represented by dots. No significant difference (*t*-test). (b) Multivariate Curve Resolution Alternating Least Squares (MCR-ALS) resolved spectral profiles of chemical components used for k-means clustering in Figure 5c. None of the resolved components are "pure" chemical compounds, but each spectrum is dominated by bands from different classes of compounds. The colors correspond to the colors of Figure 5C. Blue component spectrum: dominated by mainly carbohydrate related bands in the region of 900-1150 cm^{-1}; Red component spectrum: a mixture with clear contribution from protein bands (amide II at 1550 cm^{-1} and amide I at 1650 cm^{-1}); Yellow component spectrum: mixture of carbohydrate and protein related bands (potentially glycoproteins); Purple component spectrum: mixture with high contribution from extractives (bands at 1420 and 1630 cm^{-1}). (c) Measurements of extractable methanol in 4-day-old etiolated hypocotyls (n = 6, average ± SE). (d) Ratio of fucose, galactose and xylose in the total amount of cell wall extracted from 4-day-old etiolated hypocotyls (n = 3, average ± SE). Wild-type I represents respective control to *SYAC-GFPox* and *GFP-SYAC1ox*. Wild-type II was isolated from *syac1-5* heterozygote population. Significant differences are indicated as ***P < 0.001 (*t* test).

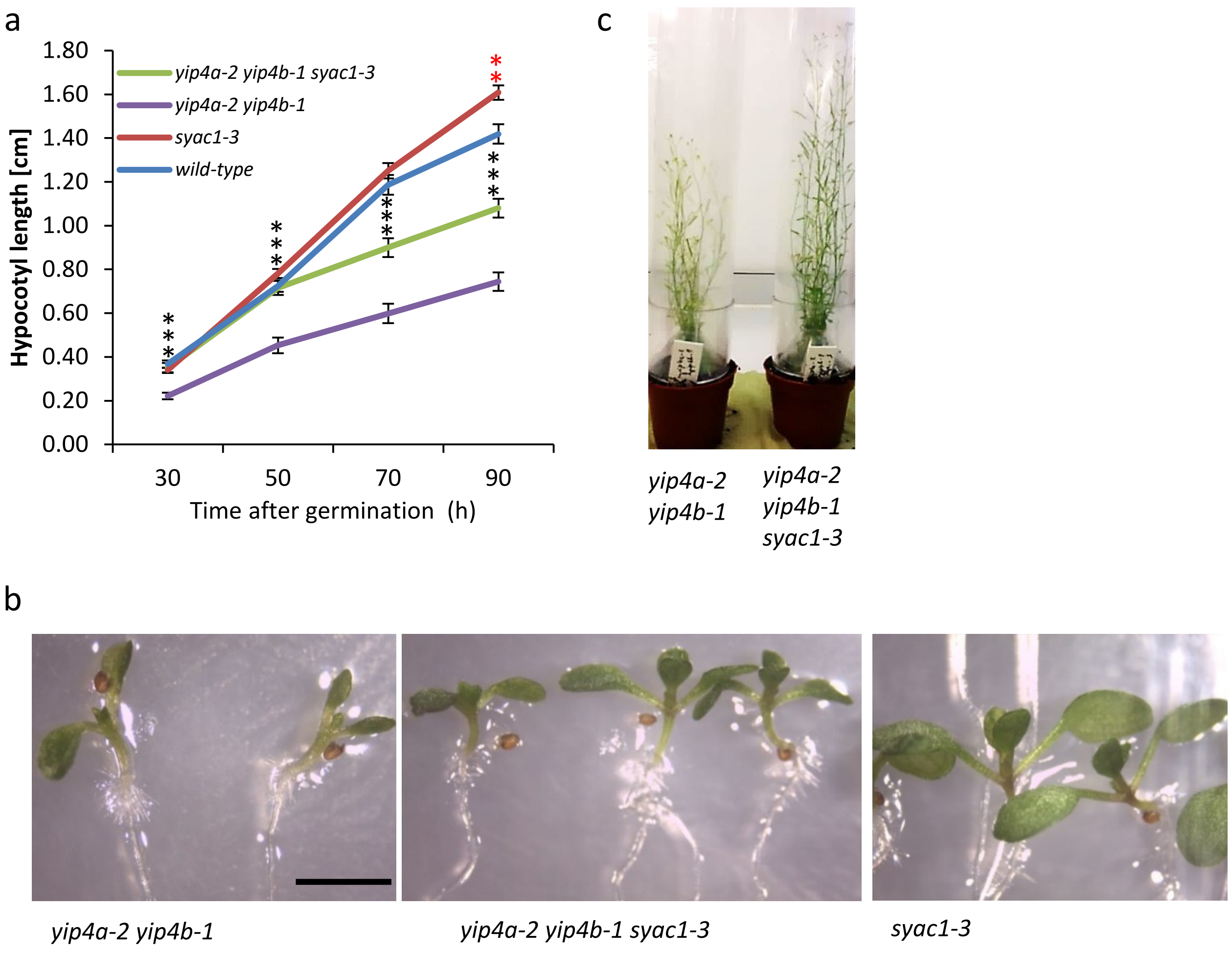

Supplementary figure 6. Loss of *syac1* expression partially recovers the *yip4a yip4b* elongation deficit. (a) Kinetics of etiolated hypocotyl growth of *syac1-3*, *yip4a yip4b* and *yip4a yip4b syac1* multiple mutant (n = 13-20 seedlings per line, average ± SE). Significant differences detected between *yip4a yip4b* and *yip4a yip4b syac1-3* indicated as ***$P < 0.001$ (*t* test) black stars; and between wild-type and *syac1-3* **$P < 0.01$ (*t* test) red stars. (b) Representative images of 7-day-old *syac1-3*, *yip4a yip4b* and *yip4a yip4b syac1-3* seedlings grown on Murashige and Skoog medium. Scale bar 500 µm. (c) Representative images of 2-month-old, *yip4a yip4b* and *yip4a yip4b syac1-3* plants.

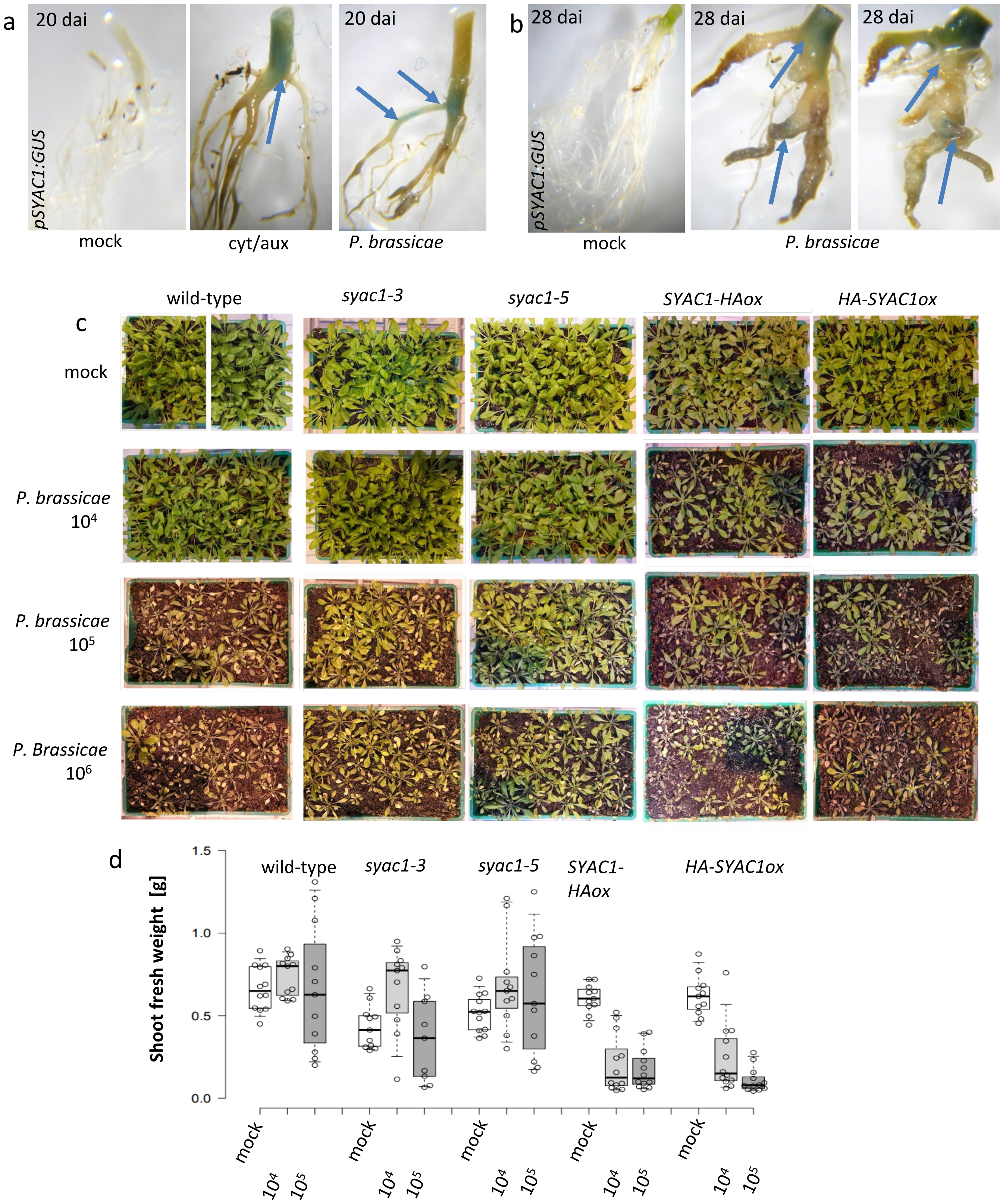

Supplementary figure 7. Loss of SYAC1 activity increases tolerance to *Plasmodiophora brassicae* infection.
(a, b) *pSYAC1:GUS* expression in *Arabidopsis* 34-day-old plants treated with 1 µM auxin and 10 µM cytokinin for 6 hours and 20 and 28 days after *P. brassicae* inoculation (dai). Inoculation was performed with 10^6 spore concentration. Blue arrows indicate GUS positive zones. (c) Shoots of wild-type, *syac1-3* and *syac1-5* and lines overexpressing *SYAC1* 28 days after *P. brassicae* inoculation. Inoculation was performed with 10^4, 10^5 and 10^6 spore concentration. 1-Naphthaleneacetic acid and N6-benzyladenine used as auxin and cytokinin, respectively. (d) Fresh weight of shoots of wild-type, *syac1-3* and *syac1-5* and lines overexpressing *SYAC1* 28 days after *P. brassicae* inoculation with control treatment (mock), 10^4 and 10^5 spore concentration. In the boxplots, center lines show the medians; box limits indicate the 25th and 75th percentiles as determined by Origin software; whiskers extend 1.5 times the interquartile range from the 25th and 75th percentiles, individual data points are represented by dots (n= 30-35 plants analyzed, one point represents average fresh weight from 2 - 3 plants).

Supplementary table 1: Sequences of primers used in the study

Gene and purpose	Name	Sequence (5'-3')
Genotyping *syac1-3* GABI-KAT 760F05	Syac1-3_LP	TGACCTTTCTTA TACTCATGCCTT
	Syac1-3_RP	TTCCACTTGTAAATGGACAACTCC
Genotyping *ech-1* SAIL 163E09	Ech-1_LP	AAACGGAAAGGGAAACACAAC
	Ech-1_RP	AGAGAAGAGTTATCGGGCTCG
Genotyping *yip4a-2* SALK_021897	Yip4a-2_LP	GTTCTTGTGGCATTGCTTCTC
	Yip4a-2_RP	TGATCTGGTTTCCACATTTCC
Genotyping *yip4b-1* SALK_129888	Yip4b-1_LP	TGTTACTTCCGC ATAAGTCGG
	Yip4b-1_RP	GCGGCTGGAGAATTCTCTATC
Genotyping T-DNA SALK	LBb1.3	ATTTTGCCGATTTCGGAAC
Genotyping T-DNA SAIL	LB3	TAGCATCTGAATTTCATAACCAATCTCGATACAC
Genotyping T-DNA GABI-KAT	RBGK.08474	ATAATAACGCTGCGGACATCTACATTTT
SYAC1 promoter cloning	pSYAC1_attB4	GGGGACAACTTTGTATAGAAAAGTTGGGGAAGACCT AGCCGTAGTT
	pSYAC1_attB1r	GGGGACTGCTTTTTTGTACAAACTTGTGATCACTTTTG GTTTTTCC
SYAC1 ORF cloning	SYAC1_attB1	GGGGACAAGTTTGTACAAAAAAGCAGGCTCCATGGA GGGCCCTTTGTTGAG
	SYAC1_attB2	GGGGACCACTTTGTACAAGAAAGCTGGGTGTCAGCA GATGCATGATACAA
SYAC1 ORF fusion with GFP tag cloning	SYAC1_attB1_Fw	GGGGACAAGTTTGTACAAAAAAGCAGGCTCCATGGA GGGCCCTTTGTTGA
	GFP_attB2_Rv	GGGGACCACTTTGTACAAGAAAGCTGGGTGTTACTTG TACAGCTCGTCCA

	SYAC1linGFP_Fw	ATTGTATCATGCATCTGCGGAGGTGGAGGTGGAGCTATGGTGAGCAAGGGCGAG
	GFPlinSYAC1_Rv	CTCGCCCTTGCTCACCATAGCTCCACCTCCACCTCCGCAGATGCATGATACAAT
SYAC1 ORF fusion with 3xHA tag cloning	SYAC1_attB1_Fw	GGGGACAAGTTTGTACAAAAAAGCAGGCTCCATGGAGGGCCCTTTGTTGA
	3xHAattB2_Rv	GGGGACCACTTTGTACAAGAAAGCTGGGTGTTATGCATAGTCCGGGACG
	SYAC1linHA_Fw	ATTGTATCATGCATCTGCGGAGGTGGAGGTGGAGCTTTCCCATATGACGTTCCA
	HAlinSYAC1_Rv	TGGAACGTCATATGGGAAAGCTCCACCTCCACCTCCGCAGATGCATGATACAAT
3xHA tag fusion with *SYAC1* ORF cloning	3xHA_attB1_Fw	GGGGACAAGTTTGTACAAAAAAGCAGGCTCCATGTTCCCATATGACGTT
	SYAC1_attB2_Rv	GGGGACCACTTTGTACAAGAAAGCTGGGTGTCAGCAGATGCATGATAC
	HAlinSYAC1_Fw	GACGTCCCGGACTATGCAGGAGGTGGAGGTGGAGCTATGGAGGGCCCTTTGTTG
	SYAC1linHA_Rv	CAACAAAGGGCCCTCCATAGCTCCACCTCCACCTCCTGCATAGTCCGGGACGTC
SYAC1 ORF fusion with mCherry tag cloning	SYAC1_attB1_Fw	GGGGACAAGTTTGTACAAAAAAGCAGGCTCCATGGAGGGCCCTTTGTTGA
	mCherry_attB2Rv	GGGGACCACTTTGTACAAGAAAGCTGGGTGTCATTTGTACAACTCATCCA
	SYAClinMCherry_Fw	ATTGTATCATGCATCTGCGGAGGTGGAGGTGGAGCTATGGTCAGCAAAGGAGAA
	mCherrylinSYAC1_Rv	TTCTCCTTTGCTGACCATAGCTCCACCTCCACCTCCGCAGATGCATGATACAAT

YIP4a ORF cloning	YIP4a_attB1	GGGGACAAGTTTGTACAAAAAAGCAGGCTCCATGTCACAAGGCGATACAGT
	YIP4a_attB2	GGGGACCACTTTGTACAAGAAAGCTGGGTGTCAATTGATGGCTATGATGA
YIP4b ORF cloning	YIP4b_attB1	GGGGACAAGTTTGTACAAAAAAGCAGGCTCCATGTCGCACAACGATACGAT
	YIP4b_attB2	GGGGACCACTTTGTACAAGAAAGCTGGGTGTCAATTAATGGCAATGATTA
YIP5b ORF cloning	YIP5b_attB1	GGGGACAAGTTTGTACAAAAAAGCAGGCTCCATGATGTCCGGCGGGAACTA
	YIP5b_attB2	GGGGACCACTTTGTACAAGAAAGCTGGGTGTCATACTTTGACATTGAAGA
ECH ORF cloning	ECH_attB1	GGGGACAAGTTTGTACAAAAAAGCAGGCTCCATGGACCCTAATAATCAGAT
	ECH_attB2	GGGGACCACTTTGTACAAGAAAGCTGGGTGTCAGACAAGGGTGAAGGCAG
KCR1 ORF cloning	KCR1_attB1	GGGGACAAGTTTGTACAAAAAAGCAGGCTCCATGGAGATCTGCACTTACTT
	KCR1_attB2	GGGGACCACTTTGTACAAGAAAGCTGGGTGTCATTCTTTCTTCATGGAGT
DSK2 ORF cloning	DSK2_attB1	GGGGACAAGTTTGTACAAAAAAGCAGGCTCCATGGGTGGAGAGGGAGATTC
	DSK2_attB2	GGGGACCACTTTGTACAAGAAAGCTGGGTGCTACTGTCCGATACTCCCCA
PHB4 ORF cloning	PHB4_attB1	GGGGACAAGTTTGTACAAAAAAGCAGGCTCCATGGGAAGTCAACAAGTAGC
	PHB4_attB2	GGGGACCACTTTGTACAAGAAAGCTGGGTGTCAACGACCAGGGTTCAGAT

CRISPR primers for *gSYAC1* amplification	CRISPR_Fw	gaatgaacttttcatttgat
	CRISPR_RV	CTTGTCTCTCTTAAGTATTCA
BASTA amplification	BASTA_Fw	atgagcccagaacgacgcccggc
	BASTA_Rv	tcagatttcggtgacgggca
SYAC1 qPCR	SYAC1_Fw	ACTTCTGGTTATGTTTGGCTCTCC
	SYAC1_Rv	ACACATATGACCACAGGCGTAAG
PP2A qPCR	PP2A_Fw	TAACGTGGCCAAAATGATGC
	PP2A_Rv	GTTCTCCACAACCGCTTGGT
EEF1A qPCR	EEF1A_Fw	CTGGAGGTTTTGAGGCTGGTAT
	EEF1A_Rv	CCAAGGGTGAAAGCAAGAAGA

Supplementary Dataset 1 and 2. (separate file)

Supplementary dataset 1.

List of proteins identified in TAP experiment with NGSTEV-SYAC1. Only retained proteins after subtraction of the background proteins according to standard procedure [98] are shown. Details on the identified peptides can be found in Supplemental data 1.

Supplementary dataset 2.

Protein identification details obtained with the LTQ Orbitrap Velos (Thermo Fisher Scientific) and Mascot Distiller software (version 2.4.1.0, Matrix Science) combined with the Mascot search engine (version 2.3.0.1, Matrix Science) using the Mascot Daemon interface and database TAIRplus [98] . Proteins and peptides headers used in the table are listed below.
