# Supplementary material for: SYNERGISTIC ON AUXIN AND CYTOKININ 1 positively regulates growth and attenuates soil pathogen resistance
Source: Nat Commun. 2020 May 1;11:2170. doi: 10.1038/s41467-020-15895-5 (PMC7195429; doi:10.1038/s41467-020-15895-5)
Supplement: Supplementary file 3 — Description of Additional Supplementary Files [file 41467_2020_15895_MOESM3_ESM.docx]

**Description of Additional Supplementary Files**

**File name:** Supplementary dataset 1

**Description:** Excel file with list of proteins identified in TAP experiment with NGSTEV-SYAC1.

**File name:** Supplementary dataset 2

**Description:** Excel file with protein identification details obtained with the LTQ Orbitrap Velos
